# Periodontal Involvement in Autoimmune Blistering Diseases: A Narrative Review

**DOI:** 10.4317/jced.64082

**Published:** 2026-07-29

**Authors:** Teresita Sarquis, Constanza Jiménez, Sven Niklander, Javier Fernández, Fernando Valenzuela, Alejandra Fernández

**Affiliations:** 1 Facultad de Odontología, Dermoral Laboratory, Universidad Andrés Bello, Santiago 8370136, Región Metropolitana Chile; 2 Department of Dermatology, Faculty of Medicine, Universidad de Chile, Santiago 8380453, Chile

## Abstract

**Background:**

Autoimmune blistering diseases (AIBDs) are characterized by autoantibody-mediated epithelial damage affecting the skin and mucous membranes. Among them, pemphigus vulgaris (PV) and mucous membrane pemphigoid (MMP) frequently involve the gingiva, most commonly presenting as desquamative gingivitis (DG). This chronic and painful gingival involvement may compromise oral hygiene and favor secondary inflammatory changes. Consequently, growing interest has focused on the periodontal status of patients with AIBDs and on associated oral microbiological findings.

**Materials and Methods:**

A narrative review was conducted using PubMed and Scopus to identify primary studies evaluating periodontal status in patients with AIBDs. Periodontal conditions were mainly assessed using conventional clinical parameters, including probing depth and clinical attachment loss, along with additional periodontal and oral hygiene indices.

**Results:**

Ten studies met the inclusion criteria. Most of them reported significantly worse periodontal status in AIBD compared to controls or DG-unaffected sites. In MMP, disease activity, duration, and DG involvement were associated with poorer periodontal parameters. Notably, adjusting for oral hygiene, as performed in only one study, rendered differences in PD and CAL non-significant, suggesting a potential confounding effect. However, the available evidence remains insufficient to support an independent link with clinically defined periodontitis. Only one study applied the 2017 World Workshop periodontitis classification, with similar conclusions. Microbiological data, limited to three MMP studies with heterogeneous methodologies, frequently detect periodontal pathogens associated with inflammatory tissue damage.

**Conclusions:**

Patients with AIBD tend to present worse periodontal status, likely due to pain limited oral hygiene and dysbiotic biofilms. These pathogens may amplify inflammation and perpetuate tissue destruction. Nevertheless, microbial profiles appear dynamic and influenced by disease activity and treatment. Recognizing this association is essential for early diagnosis and interdisciplinary management.

## Introduction

Autoimmune blistering diseases (AIBDs) are a heterogeneous group of disorders characterized by circulating autoantibodies that impair epithelial adhesion, either within the epidermis or at the dermal-epidermal junction. This disruption leads to blistering and erosions of the skin and/or mucous membranes (1 - 3). The most common AIBDs are pemphigus and pemphigoid, both significantly impact patients' quality of life (4). Pemphigus is associated with increased mortality, even with appropriate treatment, with rates estimated to be 1.7 to 3 times higher than in the general population. This is primarily due to infections and complications arising from immunosuppressive therapy (5 - 8). Pemphigoid-related deaths have also been reported, though less frequently, and are similarly linked to treatment-related complications and co-morbidities (6). Pemphigus disorders are characterized by intraepidermal blistering caused by autoantibodies targeting desmosomal cadherins. Incidence varies widely across populations, ranging from 0.6 to 32 cases per million annually (1 , 7 , 9). Pemphigus vulgaris (PV), the most prevalent subtype, accounting for approximately 70% of cases. It is primarily driven by antibodies against desmoglein 3 (Dsg3), mainly expressed in mucosal epithelium, and often targets desmoglein 1 (Dsg1), which is mainly found in the epidermis. This antigenic distribution explains the typical clinical presentation of painful oral erosions and ulcers, followed by cutaneous involvement with flaccid bullae (3 , 10). Diagnosis relies on histopathological analysis and direct immunofluorescence (3). Pemphigoid disorders involve subepidermal blistering caused by autoantibodies directed against basement membrane components. Bullous pemphigoid (BP) is the most frequent subtype, usually presenting with tense bullae on erythematous skin. In contrast, mucous membrane pemphigoid (MMP) predominantly affects mucosal surfaces, particularly the gingiva and ocular conjunctiva. Its incidence has been estimated at 1.3 - 2.0 cases per million people per year in European populations. Lesions are often painful, recurrent and may lead to complications such as vision loss. (2) Definitive diagnosis, same as for pemphigus, requires histopathology and direct immunofluorescence (2). Gingival involvement is common in both PV and MMP, typically presenting as desquamative gingivitis (DG). DG is a clinical term used to describe non-biofilm-associated gingival lesions characterized by erythema, epithelial desquamation, erosions, and occasionally ulcerations. It generally affects both the free and attached gingiva. Parlantescu et al. (2023) reported DG in 79.4% of patients with subepithelial autoimmune diseases, primarily MMP, and in 27.8% of those with intraepithelial diseases such as PV (11). Notably, DG was the only oral manifestation in 26.5% and 5.6% of these patients, respectively (11). DG is not exclusive to PV and MMP; it can also occur in other immunological conditions such as oral lichen planus, oral erythema multiforme, oral discoid or systemic lupus, among others (12). Due to the associated pain, DG may interfere with oral hygiene, leading to plaque accumulation and secondary inflammation, which can further exacerbate mucosal damage and/or periodontitis (11 , 13). Given the frequent gingival involvement in AIBDs, increasing attention has been directed toward the potential link between these conditions and other oral inflammatory diseases, such as periodontitis. Periodontitis is a chronic inflammatory disorder characterized by the progressive destruction of tooth supporting structures, initiated by a dysbiotic biofilm and modulated by local and systemic immune responses (14 , 15). Recent evidence suggests a potential association between periodontitis and AIBDs, although the precise nature of this relationship remains unclear (16 - 26). Immune dysregulation may increase the susceptible of periodontal tissues to inflammation and structural breakdown, while chronic periodontal inflammation could potentially trigger or exacerbate autoimmune responses in predisposed individuals (11 , 27 , 28). In addition, alterations in the oral microbiome have emerged as a potential contributor to the pathogenesis of AIBD. Several studies have reported a higher prevalence and diversity of proinflammatory periodontal pathogens in patients with AIBD (20 , 28 , 29). These microbial shifts may disrupt mucosal immune tolerance and promote sustained inflammatory responses. Furthermore, certain periodontal pathogens share immunoreactive sequences with Dsg3, suggesting molecular mimicry as a possible mechanism in disease initiation (27). This narrative review aims to synthesize current evidence on the relationship between periodontal involvement and AIBDs, focusing on PV and MMP, the two most common forms of AIBDs. Additionally, we also discuss recent findings on oral microbiome alterations that may contribute to the pathogenesis of these conditions.

## Materials and Methods

This narrative review was conducted following the methodological approaches proposed by Arksey and O'Malley (2005), Levac et al. (2010), and the Joanna Briggs Institute Manual for Evidence Synthesis. A protocol was prospectively registered in the Open Science Framework (DOI: 10.17605/OSF.IO/24HND). A comprehensive search of PubMed and Scopus was performed between May and June 2025, restricted to studies published in English or Spanish from 2005 to 2025. Eligible criteria included patients with pemphigus or pemphigoid, in whom periodontal status was assessed. Exclusion criteria were case reports, secondary studies, in vitro or animal research, conference abstracts and articles not indexed in PubMed. Titles, abstracts, and full texts were screened against predefined eligibility criteria. Data on study characteristics, periodontal and microbiological parameters, and main findings were extracted. Due to the heterogeneity among studies, results were synthesized descriptively using both narrative and tabular formats, without quantitative pooling. The detailed search strategy is provided in (Supplement 1) (http://www.medicinaoral.com/medoralfree01/aop/jced_64082_s01). - Overview of Included Evidence The initial search retrieved a total of 338 records (PubMed: 85; Scopus Advanced: 253). After removing duplicates, 324 articles were screened for eligibility. Of these, 10 studies met the inclusion criteria and were included in the final analysis. The included studies were published between 2006 and 2024. Most were conducted in Italy (n = 4), followed by Turkey (n = 2), USA (n = 2), India (n = 1) and France (n = 1). Five studies had a case-control design and five were cross-sectional. A total of 464 participants were evaluated, including 264 patients diagnosed with AIBDs and 200 control subjects. Twelve patients were evaluated twice in two separate studies by Lo Russo et al. (2010 and 2014). Among the patients, 133 had PV, 100 had MMP, 4 had BP and 27 had Oral Lichen Planus (OLP). In most cases, the diagnosis was confirmed both clinically and histologically. Details from each study are summarized in Table 1.

Table 1

- Clinical Periodontal Parameters Assessed All included studies evaluated periodontal status using various clinical parameters, which were categorized according to the primary and complementary criteria of periodontitis, as defined by international consensus guidelines (15) (Table 2).

Table 2

Among the primary criteria, probing depth (PD) was the most frequently evaluated parameter, reported in all studies. Clinical attachment level (CAL), bleeding on probing (BOP), and full-mouth bleeding score (FMBS) were also commonly assessed, whereas marginal bone loss (MBL) was reported in only one study. Secondary criteria were less frequently assessed. Tooth loss attributable to periodontitis was reported in seven studies, tooth mobility in six, and both furcation involvement and gingival recession (REC) in four. None of the studies reported suppuration, halitosis, or radiographic evidence of vertical or horizontal bone defects. Other parameters assessed were plaque index (PI), evaluated in six studies, and gingival index (GI), in three. Several studies also applied composite or standardized measures to assess different aspects of patient's condition: - Oral hygiene: Plaque index (19 - 22 , 24) Full Mouth Plaque Score (FMPS) (17 , 18 , 23 , 25) Daily frequency of tooth brushing (16) Domiciliary Oral Hygiene (DOH) scores (17) Plaque control record (20) - Disease severity: Clinical Severity Score (CSS) (16 , 19) Autoimmune Bullous Skin Disorder Intensity Score (ABSIS) (21) - Symptoms and quality of life: Visual Analogue Scale (VAS) (21) Oral Health Impact Profile 14 (OHIP-14) (21) Generic quality of life questionnaires (SF-36) (21) - Clinical Findings Main findings are summarized in Table 3.

Table 3All five case control studies reported significantly worse periodontal conditions in AIBD patients compared to healthy controls, including higher GI and, in some cases, increased PI, REC, BOP/FMBS, PD, CAL, tooth loss, or radiographic bone loss (p < 0.05). No significant differences were reported in furcation involvement or mobility scores (p > 0.10) (16 , 17 , 19 , 22 , 24). Tricamo et al. (2006) also reported that patients with active MMP had higher PI than those in remission (P<0.03), and that longer disease duration was associated with increased REC (P<0.003) and furcation involvement (P<0.03). (24) Among the five remaining studies, four reported poorer periodontal status in individuals with AIBD, either in comparison to healthy controls or those with plaque-induced gingivitis, or within AIBD cohorts comparing DG-affected and unaffected sites. (18 , 20 , 21 , 23) In contrast, one cross-sectional study comparing DG-affected and unaffected sites within the same patients reported no significant clinical differences (p > 0.106). (25) However, periodontal parameters assessed varied across studies, and formal diagnostic criteria for periodontitis were not consistently applied. Oral hygiene behaviors were assessed in all studies, although using different parameters, thus yielding inconsistent results. Six studies reported poorer hygiene, based on domiciliary scores (17), FMPS (17 , 18 , 23) and PI (19 - 21). Notable, Arduino et al. (2011) was the only study that perform a multivariate adjustment for this variable; they observed that differences in PD and CAL lost significance after adjusting for FMPS (p = 0.3) and reported a strong positive correlation between FMPS and both PD (R=0.6, P<0.001) and FMBS (R=0.7, P<0.0001), suggesting that oral hygiene may account for the observed differences (18). In contrast, other studies found no significant differences in PI (22 , 24), FMPS (25), or brushing frequency (16). Some studies employed multivariate models to assess whether periodontal changes were independently associated with disease severity or activity. In MMP patients, Lo Russo et al. (2010) reported a negative association between DG and shallow probing depth (< 4 mm) (p = 0.004), along with a positive association with moderate depths (4-6 mm) (p = 0.0005), suggesting a possible link between DG lesions and moderate pocket formation. (25) In a subsequent study, Lo Russo et al. (2014) found significant correlations between CAL, PD, and REC in DG positive sites only (p < 0.019), supporting a site-specific effect of mucosal lesions. (23) Regarding disease severity in PV patients, Thorat et al. (2010) found no independent association between PD, CAL, or treatment duration and disease severity (p > 0.05). (19) Akman et al. (2008) also reported no correlation between Community Periodontal Index of Treatment Needs (CPITN) and CSS (p > 0.077). (16) - Microbiological Findings Only three studies reported microbiological findings (Table 4). Table 4

**Figure 1:**
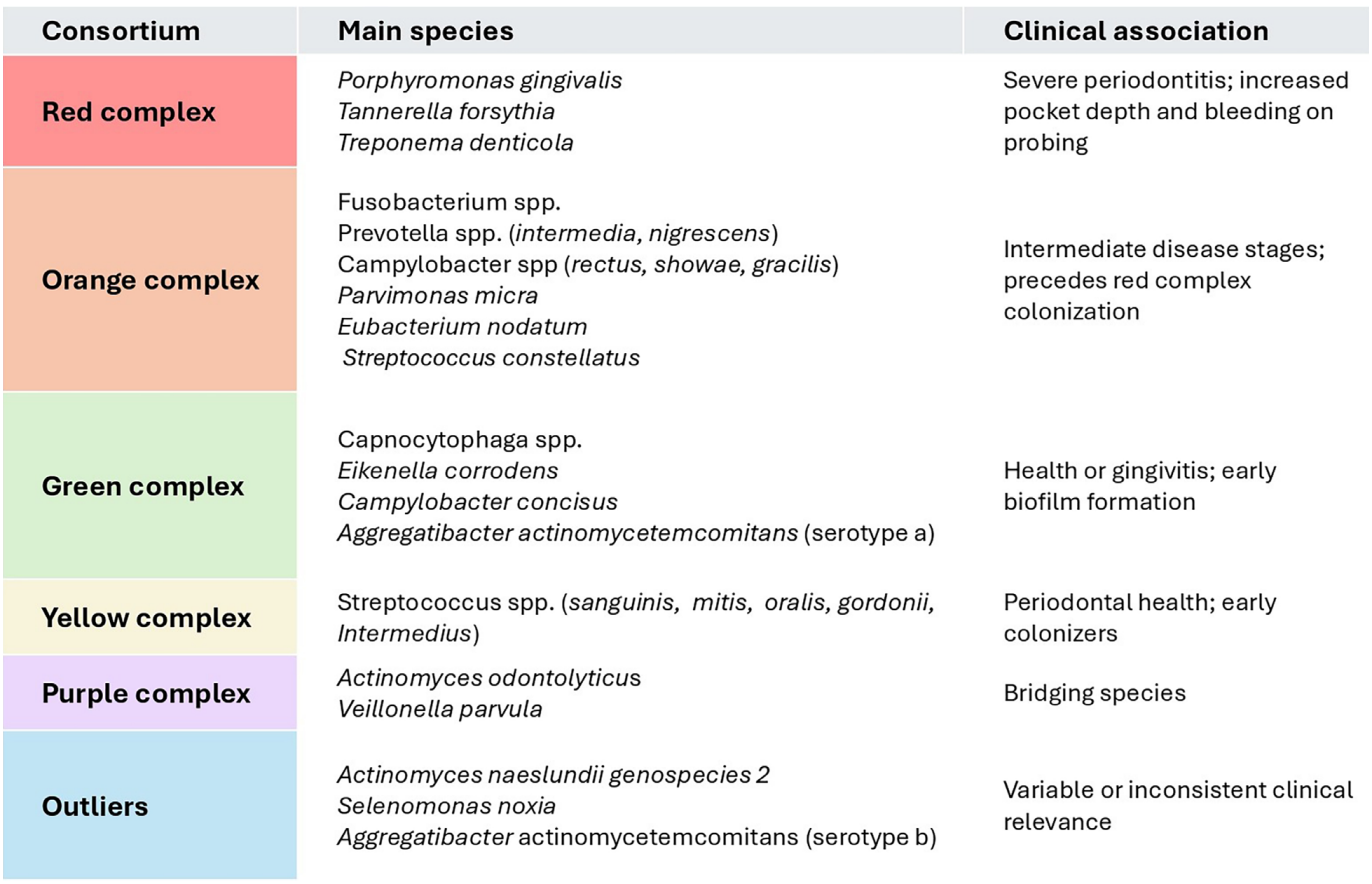
Periodontal bacterial consortia according to the classification proposed by Socransky et al. (1998) [39,40].

To facilitate comparison, these data were grouped by bacterial consortia. The red complex is most strongly associated with periodontitis, whereas the orange complex represents an intermediate stage that often precedes red complex colonization. Details of bacterial consortia are provided in Figure 1.

1

Microbial samples were obtained from gingival or subgingival sites in patients with AIBD, although sampling methods and lesion definitions vary across studies. Lo Russo et al. (2014) also included DG-unaffected sites (23) and Arduino et al. (2017) included controls with plaque-induced gingivitis and no history of AIBD. (18) All studies evaluated periodontal species from the red and orange complex, consistently detecting at least one red complex species and multiple orange complex species in most patients, while species from other complexes were variably assessed. Lo Russo et al. (2014) analyzed subgingival plaque samples from both DG positive and DG negative sites in patients with MMP. They found that bacterial co-occurrence patterns varied according to lesion status. In DG positive sites, strong correlations were observed among red and orange complex species, including P. gingivalis, T. denticola, T. forsythia, P. intermedia, F. nucleatum and P. micra (p < 0.01). In contrast, DG negative sites showed stronger correlations with T. denticola, T. forsythia, P. intermedia, and P. gingivalis (p < 0.00002). The presence of A. actinomycetemcomitans was positively correlated with the absence of lesions (r = 1, p = 0) (23). Arduino et al. (2017) collected subgingival plaque samples from patients with OLP or MMP, as well as from controls with plaque-induced gingivitis. They reported a significantly higher prevalence of A. actinomycetemcomitans, E. corrodens, and F. nucleatum in patients with DG compared to those with gingivitis (p < 0.03). However, in multivariate analysis, only E. corrodens remained significantly associated with DG (adjusted OR = 12.78). No microbial differences were observed between the OLP and MMP subgroups (p > 0.08) (18). Ejeil et al. (2024) collected subgingival biofilm samples from the deepest periodontal pockets in patients with MMP with gingival expression. They identified three dysbiotic bacterial consortia dominated by red and orange complex species, including P. gingivalis, T. forsythia, T. denticola, P. intermedia, P. micra, F. nucleatum, C. rectus, and P. nigrescens. These consortia were found in varying proportions of patients, but no significant association was observed between any consortium and severe periodontitis (p > 0.05). Other species from the green, yellow, blue, and purple complexes, as well as C. albicans, were also frequently detected (20). None of the studies demonstrated a clear or consistent association between microbiota profiles and either the presence or severity of periodontitis. - Clinical and Biological Interactions Between AIBD and Periodontitis Current evidence suggests a trend toward poorer periodontal parameters in patients with AIBD (16 - 24). In patients with MMP, worse periodontal status has been linked to active disease and longer duration, while site-specific associations with DG lesions have also been reported (23 - 25). Conversely, no consistent independent association has been found between periodontal parameters and disease severity in PV (16 - 19). Only one study applied the 2017 World Workshop classification of periodontitis (21) and reached similar conclusions, suggesting that case definition changes do not alter the observed association with AIBD. However, methodological heterogeneity and the infrequent adjustment for oral hygiene, a key potential confounder, render the association between AIBDs and periodontitis inconsistent and preclude definitive conclusions. The poorer periodontal status in PV and MMP patients, primarily reflected in worse PD, CAL, BOP and FMBS, may be partially explained by the pain and discomfort caused by DG involvement, which restricts effective oral hygiene and may reduce dental care, favoring plaque accumulation and gingival inflammatio (16 , 17 , 19 - 22 , 24 , 25) Furthermore, autoimmune-mediated gingival degradation may also increase the exposure of antigenic epitopes and facilitate pathogen translocation, potentially accelerating periodontitis progression (16). Microbiological data provides further insight into the association between AIBD and periodontal disease, particularly in MMP. In these patients, subgingival biofilms showed consistently higher prevalence of P. gingivalis, T. denticola, T. forsythia, P. intermedia, F. nucleatum, and P. micra (18 , 20 , 23), pathogens commonly associated with periodontal diseases. P. gingivalis degrades epithelial adhesion molecules and immune regulators. (30) T. denticola disrupts epithelial junctions and neutrophil function, facilitating deeper bacterial invasion (31). T. forsythia secretes proteases and lipoproteins that can stimulate IL-6, IL-8 and TNF- secretion, driving periodontal tissue destruction (32). F. nucleatum acts as a bridging organism, supporting biofilm maturation and inducing bone resorption (33), while P. micra reinforces dysbiosis through epithelial adhesion and synergistic interactions (34). Together, these pathogens may accelerate periodontal breakdown and contribute to immune dysregulation in AIBD (30 , 32 , 33 , 35). Arduino et al. (2017) reported that, after multivariate adjustment, only E. corrodens prevalence remained significantly associated with DG compared with plaque-induced gingivitis (18). Although this species belongs to the green complex, usually associated with health or gingivitis, it may promote matrix degradation and bone resorption via fibroblast activation (36). In this review, no microbiological data was available for patients with PV. The literature describes heterogeneous oral microbiome profiles, with inconsistent findings regarding bacterial diversity and dominant taxa (28 , 29 , 37). These inconsistencies likely reflect differences in study design, taxonomic resolution, disease phase, and treatment. Beyond taxonomic shifts, microbial interactions with host immunity have been proposed. Petruzzi et al. (2022) demonstrated peptide homology between Dsg3 and bacterial proteomes, particularly E. corrodens, F. nucleatum, and T. forsythia, supporting molecular mimicry as a possible pathogenic pathway (27).

## Results

sfasjbdgkjbva

## Discussion

asdgasg

## Conclusions

Overall, AIBD patients tend to present worse periodontal status, likely resulting from impaired oral hygiene and the presence of dysbiotic microbiota. These pathogens may amplify local immune responses, creating a vicious cycle of inflammation and tissue destruction (17 , 19 - 21 , 25). Microbial shifts may act as both triggers and consequences of disease activity or immunosuppressive therapy (38). However, gingival indices must be interpreted cautiously, as autoimmune lesions can mimic plaque-induced inflammation (17 , 21 , 22). Moreover, microbial profiles appear dynamic and influenced by disease activity, treatment, and methodological variability. Further longitudinal and integrative studies, incorporating bacterial, viral, and fungal communities, are needed to clarify causality.

## Figures and Tables

**Table 1:** Studies characteristics. [16-25].

Authors (Year)	Country	Study Design	Objective	Population	Exclusion Criteria
Tricamo et al. (2006)	USA	Case control	To evaluate the possible impact of gingival pemphigoid lesions on the human periodontium.	20 patients with clinical and histological diagnosis of MMP (12 female, 8 males, mean age 59.5 ± 10.2); 20 age, gender and smoking matched controls without MMP	Patients with less than 20 teeth; History of surgical periodontal therapy; Pregnant females; History of diabetes mellitus; Uncontrolled cardiovascular disease or active infectious disease.
Akman et al. (2008)	Turkey	Case control	To assess periodontal status in PV patients and whether it correlates with disease severity.	20 patients with clinical, histological and immunofluorescence diagnosis of PV (9 female, 11 males, mean age 42.9 ± 9.8 years); 22 healthy controls without any inflammatory disorders or systemic treatments (8 female, 14 males, mean age 40.5 ± 12.1 years)	Not reported
Schellinck et al. (2009)	USA	Case control	To evaluate the changes in periodontal status in a patient with MMP after a period of 5 years.	10 patients with clinical, histological and immunofluorescence diagnosis of MMP (mean age 76 years); 10 controls with no history of MMP, matched for age, gender and smoking history (mean age 75 years)	Pregnant females; History of diabetes mellitus; Uncontrolled cardiovascular disease; Active infection disease; Control patients who subsequently developed gingival MMP or other conditions causing DG.
Thorat et al. (2010)	India	Case control	To assess the periodontal status of patients with PV compared to healthy individuals.	50 patients with clinical, histological and immunofluorescence diagnosis of PV, under treatment for >2 years (24 female, 26 male, mean age 35.2 ± 7.8); 50 healthy controls, without any inflammatory disorders and not under any systemic treatment (24 female, 26 male, mean age 37.0 ± 8.4)	Patients who had received any periodontal therapy within the last 6 months; Patients with any systemic disease other than PV.
Arduino et al. (2011)	Italy	Case control	To compare the periodontal status of patients with MMP and healthy controls.	29 patients with clinical, histological and immunofluorescence diagnosis of MMP (25 female, 5 male, mean age 54.5 ± 16.2, smoking current 3); 30 controls with no history of DG related to MMP (20 female, 10 male, mean age 51.7 ± 2.4 years, smoking current 5)	History of previous and/or current treatment for desquamative gingival lesions; History of previous periodontal therapy; Less than 18 teeth; Pregnancy; Patients with history of diabetes mellitus and uncontrolled cardiovascular diseases.
Lo Russo et al. (2010)	Italy	Cross-sectional	To evaluate the potential impact of DG lesions on plaque related periodontal attachment loss and pocket formation.	12 patients with clinical diagnosis of DG: 8 OLP; 4 MMP (standardized diagnostic workflow Lo Russo et al, 2008) (10 female, 2 males, mean age 59.66 years, none smoking current)	History of previous and/or currenttreatment for desquamative gingival lesions; Inclusion in previous and/or current oral hygienemaintenance programs; Previous and/or currenttreatments for plaque-related periodontal disease; Pregnancy
Lo Russo et al. (2014)	Italy	Cross-sectional	To evaluate the relation between clinical and microbiological parameters and the presence of DG lesions.	12 patients: 8 OLP; 4 MMP (standardized diagnostic workflow Lo Russo et al, 2008, confirmed by histo and/or immunopathologic examination)(OLP: 7 female, 1 male, mean age 60.6 years. MMP: 3 female, 1 male, mean age 57.7 years)	Not reported (same patients as Lo Russo et al. 2010)
Arduino et al. (2017)	Italy	Cross-sectional	To investigate the prevalence of 11 periodontopathogen microorganisms in DG patients and compare it with the microbiological profile of controls with plaque-induced gingivitis.	33 patients with histological diagnosis of OLP (N = 19) or MMP (N = 14), with gingival lesions related to the disease (mean age 68.85 years);33 patients with plaque induced gingivitis, without history of DG, OLP or MMP, in good general health, with PD ≤3mm, no attachment loss and BOP ≥20%.	Histological signs of dysplasia; Treatment for OLP and MMP within the last 3 months; Antibiotic use in the last 3 months: Periodontal therapy in the last 6 months; Smoking habits; Pregnancy or lactation.
Bilgic et al. (2020)	Turkey	Cross-sectional	To evaluate the association between oral health related quality of life (OHRQoL) and disease severity in patients with AIBD.	67 patients with AIBD (No diagnostic criteria were mentioned) (63 PV, 4 BP. 40 female, 24 male, mean age 53.48 ± 15.27);35 healthy controls without AIBD or any inflammatory disorder (20 female, 15 males, mean age 54.86 ± 7.1 years)	Not reported
Ejeil et al. (2024)	France	Cross-sectional	To characterize the subgingival microbiota in patients with gingival MMP and explore its potential association with clinical features of the bullous disease and periodontal status.	23 patients with acute gMMP (with formal diagnosis by hospital dermatologist), untreated or recurrent persistent despite medical treatment (17 female, 7 male, mean age 67.4 ± 10)	The existence of antibiotic or antifungal treatment during 3 months before the study; The existence of nonsurgical periodontal treatment in the 3 months before the study; The patients that refuse to participate in the study and persons subject to legal protection.

Mucous Membrane Pemphigoid (MMP), Plaque index (PI), Gingival index (GI), Bleeding on probing (BOP), Probing depth (PD), Clinical attachment level (CAL), Gingival recession (REC), Pemphigus vulgaris (PV), Community Periodontal Index of Treatment Needs (CPITN), Clinical Severity Score (CSS), Full mouth gingival bleeding score (FMBS), Oral hygiene domiciliary scores (DOH), Full mouth plaque score (FMPS), Visual Analogue Scale (VAS), oral health related quality of life (OHRQoL), Autoimmune bullous disease (AIBD), Bullous Pemphigoid (BP), Aggregatibacter actinomycetemcomitans (Aa), Fusobacterium nucleatum (Fn), Eikenella corrodens (Ec), Autoimmune Bullous Skin Disorder Intensity Score (ABSIS), Oral Health Impact Profile with 14 items (OHIP-14), Generic quality of life questionnaires (SF-36), Prevotella intermedia (Pi), Treponema denticola (Td), Tannerella forsythia (Tf), Porphyromonas gingivalis (Pg), Parvimonas micra (Pm), Campylobacter rectus (Cr), MMP with gingival expression (gMMP).

**Table 2:** Overview of periodontal parameters assessed in the included studies [16-25].

Periodontal parameters assessed			Nº of studies reporting
Primary Criteria	Loss of Periodontal Supporting Tissue	Clinical Attachment Level (CAL)	9
		Marginal bone loss (MBL) radiographically assessed	1
	Periodontal Pocket Formation	Probing depth (PD)	10
	Gingival inflammation	Bleeding on probing (BOP) or full-mouth bleeding score (FMBS)	9
Secondary Criteria	Suppuration		0
	Halitosis		0
	Furcation involvement		4
	Tooth mobility		6
	Tooth loss (attributable to periodontitis)		6
	Gingival recession (REC)		4
	Radiographic evidence of vertical and/or horizontal bone defects		0
Others	Plaque Index (PI)		5
	Gingival Index (GI)		3

Clinical attachment level (CAL), Marginal bone loss (MBL), Probing depth (PD), Bleeding on probing (BOP), Full mouth gingival bleeding score (FMBS), Gingival recession (REC), Plaque index (PI), Gingival index (GI).

**Table 3:** Periodontal assessment parameters and main results [16–25].

Authors (Year)	Periodontal Parameters evaluated	Diagnostic criteria for periodontitis	Other Parameters Evaluated	Key Findings associated with the research question	Association reported	Other Key Findings
Tricamo et al. (2006)	PI, GI, BOP, PD, CAL, REC, mobility, furcation involvement, number of missing teeth	Case definition for severe periodontitis according to Page & Eke framework.	Time since diagnosis, patients on treatment.	MMP patients showed significantly higher GI scores compared to matched controls (1.82 vs 0.89, P<0.0001).No significant differences were found in PI, BOP, PD, CAL, REC, mobility, furcation involvement, number of missing teeth or periodontitis diagnosis (P>0.376).	Not reported	Active MMP cases had higher PI than cases in remission (P<0.03). Longer disease duration is linked to more REC (P<0.003) and furcation involvement (P<0.03).
Akman et al. (2008)	CPITN (including BOP and PD)	Case definition for periodontal disease according to the CPITN Index, corresponding to codes 3 or 4.	Oral hygiene habits, presence of prosthesis, presence of gingival lesions, number of carious teeth, CSS, treatment.	The mean CPITN score was significantly higher in patients with PV (2.8 ± 0.7) than controls (4.2 ± 2.7) (P < 0.001). Nevertheless, there was no significant difference in CPITNaccording to CSS (P=0.4)	Logistic regression analysis of the factor affecting the severe CPITN and the factor affecting the higher CSS revealed no significant difference (P>0.077).	The number of carious teeth was significantly higher in PV patients (P=0.007).No significant differences between groups regarding presence of gingival lesions, brushing frequency or prosthesis use (P=0.252).
Schellinck et al. (2009)	PI, GI, BOP, PD, facial and lingual recession, CAL, mobility, furcation involvement, number of missing teeth	Case definition for severe periodontitis according to Page & Eke framework.	Time since diagnosis, disease activity, treatment.	At both time periods, MMP patients had significantly higher GI (P<0.04) and lingual recessión (P<0.01) compared to controls. No significant differences were found in PI, BOP, PD, CAL, facial recession or periodontitis classification (P>0.10).	Not reported	Evaluating the overall change in 5 years, both groups showed a significant increase in CAL (P=0.000), facial recession (P=0.003) and lingual recession (P=0.01), but the difference in change between groups was not significant in any of the clinical parameters (P>0.43).
Thorat et al. (2010)	PI, PD, CAL, FMBS, Radiographic bone loss	Not reported	CSS, treatment, oral lesions, disease location, daily tooth brushing.	PV patients had significantly higher PD (4.51 vs 3.84, P<0.05), CAL loss (3.16 vs 2.44, P<0.05) and PI (41.6 vs 24.8, P< 0.001) than controls. Also they had more radiographic bone loss (86.66%) than healthy controls (46.66%).	Logistic regression confirmed that age, gender, PD, CAL, and treatment duration were not independently associated with PV severity (P>0.05).	Intra and inter arch comparison in the PV group showed higher PD and CAL in the upper arch compared to the lower arch of PV patients and to controls (P < 0.0394). The CAL difference in the lower arch between groups was not statistically significant (P > 0.05).
Arduino et al. (2011)	FMBS, PD, REC, CAL, mobility score, molar furcation involvement, number of missing teeth	Case definition for severe periodontitis according to a modified Page & Eke framework.	FMPS, mean delay diagnosis, DOH.	MMP patients had significantly higher PD (0.8 ± 0.2mm, 95% CI 0.3-1.3), CAL (1.3 ± 0.4mm, 95% CI 0.4-2.2), FMPS (41 ± 6.2%, 95% CI 28.7-53.4), FMBS (16.2 ± 6.6%, p5% CI 3.0-29.4), and tooth loss (2 ± 1 teeth, 95% CI 1-3) than controls (P<0.01 for all). No difference in furcation involvement and mobility score (data not shown).Only 2 MMP patients and 1 control met the criteria for periodontitis.	Differences in PD and CAL remained after adjusting for demographics covariates (PD: 3.2 vs 2.3, P<0.0001, F=5.1, R2=0.3. CAL: 2.8 vs 1.5, P=0.006, F=3.0, R2=0.1), but disappeared when adjusting for all covariates including FMPS (PD: 3.0+0.2mm vs 2.8+0.22, P=0.5, F=9.2, R2=0.5. CAL: 2.3+0.3mm vs 2.8+0.3mm, P=0.3, F=11.6; R2=0.5). Linear correlation analysis confirmed a strong positive correlation between FMPS with both PD (R=0.6, P<0.001) and FMBS (R=0.7, P<0.0001).	MMP patients had poorer oral hygiene routines than controls (P<0.0001).
Lo Russo et al. (2010)	FMBS, PD, CAL, number of missing teeth	Not reported	FMPS, symptoms, disease location (sites affected by DG)	No significant differences in median PD, CAL, FMPS, or FMBS were observed between affected and unaffected sites, in both OLP and MMP patients (P>0.106).	When comparing DG affected and unaffected sites in MMP patients, a negative association was found between DG lesions and PD < 4 mm (OR 0.47, 95% CI: 0.27–0.79; P = 0.004), and a positive association with PD between 4–6 mm (OR 2.68, 95% CI: 1.49–4.81; P = 0.0005). No significant association was observed with PD > 6 mm (P = 0.805).	DG lesions were significantly associated with PD >6 mm only in OLP (P=0.0291).
Lo Russo et al. (2014)	FMBS, PD, CAL, REC	Not reported	FMPS, disease location, Microbiological data by PCR	In MMP cases, gingival sites with DG lesions showed significantly higher REC (1.69mm vs 1.33mm, P=0.00001), CAL (4.02mm vs 3.79mm, P=0.006), and FMPS (0.99 vs 0.91, P=0.049) than DG negative sites.	In MMP cases, DG positive sites showed a significant correlation between CAL and PD (r=0.61, p=0.014, r²=0.38) as well as between CAL and REC (r=0.59, p=0.019, r²=0.35). In contrast, there was no significant correlation between CAL and PD in sites where MMP lesions were absent (data not shown).	The presence of Aa was associated with absence of DG lesions; other microbial correlations varied by lesion status.
Arduino et al. (2017)	FMBS, PD, REC, CAL, tooth mobility, molar furcation involvement, number of missing teeth.	Periodontitis defined according to the 1999 International Workshop classification	FMPS, microbiological data by PCR	DG patients showed significantly higher FMPS, FMBS, CAL, PD and number of missing teeth than controls (P < 0.001).	Not reported	Significantly higher prevalence ofAa, Ec, and Fn in DG (P<0.03). Only Ec remained significantly associated after adjustment (OR=12.78).
Bilgic et al. (2020)	Oral health was evaluated in 49.1% of active patients (13 patients):Number of natural teeth, PI, GI, PD, CAL	Not reported	ABSIS, OHIP-14, SF-36, self-reported oral health status, VAS due to oral lesions.	Patients with AIBD had significantly higher PI and GI (PI: 2.19 ± 0.57, GI: 1.91 ± 0.42) compared to healthy controls (PI: 1.67 ± 0.40, GI: 1.53 ± 0.27. P < 0.001). No significant differences were found in PD, CAL, or number of natural teeth (P>0.091).No correlation was observed between OHIP-14 and periodontal parameters or number of natural teeth (P>0.05).	Not reported	Patients with active disease had significantly higher OHIP-14 scores (42.28 ± 13.66) compared to those with inactive disease (29.08 ± 12.25; P = 0.004), and these were positively correlated with pain levels (mean pain score 6.33 ± 2.78; r = 0.409, P = 0.013). Additionally, patients with a significant disease course reported higher OHIP-14 scores (45.18 ± 15.08) compared to those with a moderate course (36.09 ± 9.73; P = 0.010).
Ejeil et al. (2024)	PI, BOP, Number of missing teeth, PD, CAL, tooth mobility, degree of interdental alveolysis.	Case definition for periodontitis according to the AAP/EFP classification system for periodontal diseases	Disease duration, specific medical treatment, plaque control record	All patients had a consistently elevated PI (48–100%, mean >83%) and BOP (20–98%, mean >54%), as well as a high prevalence of periodontal pathogens. Eleven patients had severe periodontitis, eleven non-severe periodontitis, and one severe plaque-induced gingivitis.	Not reported	Longer gMMP duration (≥1 year) was significantly associated with greater severity of erosive gingivitis (p=0.009) and periodontitis (p=0.04).However, no significant associations were found between the severity of erosive gingivitis or periodontitis and age, gMMP specific treatment, extraoral lesions, comorbidities, or extent of gingival involvement.A bacterial group including Pg, Tf, Td, Pi, Pm, Fn, and Cr tended to be more prevalent in severe periodontitis, but the association was not statistically significant (P>0.05).

Mucous Membrane Pemphigoid (MMP), Plaque index (PI), Gingival index (GI), Bleeding on probing (BOP), Probing depth (PD), Clinical attachment level (CAL), Gingival recession (REC), Pemphigus vulgaris (PV), Community Periodontal Index of Treatment Needs (CPITN), Clinical Severity Score (CSS), Full mouth gingival bleeding score (FMBS), Oral hygiene domiciliary scores (DOH), Full mouth plaque score (FMPS), Visual Analogue Scale (VAS), oral health related quality of life (OHRQoL), Autoimmune bullous disease (AIBD), Bullous Pemphigoid (BP), Aggregatibacter actinomycetemcomitans (Aa), Fusobacterium nucleatum (Fn), Eikenella corrodens (Ec), Autoimmune Bullous Skin Disorder Intensity Score (ABSIS), Oral Health Impact Profile with 14 items (OHIP-14), Generic quality of life questionnaires (SF-36), Prevotella intermedia (Pi), Treponema denticola (Td), Tannerella forsythia (Tf), Porphyromonas gingivalis (Pg), Parvimonas micra (Pm), Campylobacter rectus (Cr), MMP with gingival expression (gMMP)

**Table 4:** Microbiological data in studies reporting oral microbiota [18,20,23].

Authors (Year)	Population	Sample Type and Collection Method	Microbiological Method	Bacteria Assessed	Main Results
Lo Russo et al. (2014)	12 patients with AIBD (8 OLP, 4 MMP)	Subgingival plaque samples from four sites per patient (two DG negative and two DG positive), collected with sterile paper points after cleaning and isolation.	Real-time PCR (Meridol Perio Diagnostics® kit).Results expressed as relative percentage of each species.	A. actinomycetemcomitans, P. gingivalis, T. forsythia, T. denticola, F. nucleatum, P. intermedia.	In MMP patients, specific bacterial associations varied according to lesion status. In DG-positive sites, A. actinomycetemcomitans was significantly correlated with P. gingivalis (r = 0.63, p = 0.01). Additional inter bacterial associations were observed among red and orange complex species, including F. nucleatum, T. denticola, T. forsythia, and P. intermedia (p < 0.01). In DG-negative sites, A. actinomycetemcomitans was positively associated with the absence of gingival lesions (r = 1, p = 0), and P. gingivalis showed a strong correlation with P. intermedia (r = 0.87, p = 0.00002). Inter-bacterial correlations were generally stronger in DG-negative sites, particularly among T. denticola, T. forsythia, and P. intermedia (r ≥ 0.99, p < 10⁻²⁵).
Arduino et al. (2017)	33 patients with DG (19 OLP, 14 MMP) and 33 matched controls with plaque induced gingivitis and no history of AIBD; all with PD ≤ 3 mm.	Subgingival plaque samples from four inflamed sites (BOP +, PD ≤3 mm), one per quadrant. In DG patients, sites also had clinical signs of DG. They were collected using two sterile paper points per site after supragingival cleaning, isolation, and drying.	Semi-quantitative PCR (DNA-strip hybridization assay). Bacterial load categorized from 0 (<10³) to 4 (>10⁷ cells), with 0 indicating absence.	A. actinomycetemcomitans, P. gingivalis, P. intermedia, T. forsythia, T. denticola, P. micra, F. nucleatum, C. rectus, E. nodatum, E. corrodens, Capnocytophaga spp.	F. nucleatum, A. actinomycetemcomitans, and E. corrodens showed significantly higher prevalence in DG than plaque induced gingivitis patients (p < 0.03). In multivariate models, only E. corrodens remained significantly associated with DG (adjusted OR = 12.78). No microbiological differences were found between OLP and MMP subgroups (p > 0.08).
Ejeil et al. (2024)	15 patients with gMMP	Subgingival plaque from the three deepest pockets (two sterile paper points per pocket).	Quantitative real-time PCR (SYBR Green, Rotor-Gene Q thermocycler) with species-specific 16S rRNA primers; Results expressed as absolute and relative quantities. Associations >0.01% relative abundance analyzed.	C. albicans and main periodontal pathogens from Socransky’s complexes, including A. actinomycetemcomitans, P. gingivalis, T. forsythia, T. denticola, F. nucleatum, P. intermedia, P. nigrescens, C. rectus, P. micra, and others.	Fourteen patients showed abundant plaque levels (≥10¹⁰), regardless of gMMP duration, treatment, erosive gingivitis, or periodontitis severity; one had slightly less (9.6×10⁹). Red complex bacteria (P. gingivalis, T. forsythia, T. denticola) were detected at ≥0.01% in all patients for at least one species, with all three present on 4/15. Orange complex bacteria (P. intermedia, P. micra, F. nucleatum, C. rectus, P. nigrescens) were consistently found, with at least three species ≥0.01% in all patients. Three dysbiotic bacterial consortia were identified: consortium 1 (100%), consortium 2 (60%), and consortium 3 (26%). No significant association was found between severe periodontitis and consortium 3 (p = 0.05) or with medical variables. Other complexes (green, yellow, blue, purple) were also frequently detected, and C. albicans was present in 5 patients.

Aggregatibacter actinomycetemcomitans (A. actinomycetemcomitans), Porphyromonas gingivalis (P. gingivalis), Tannerella forsythia (T. forsythia), Treponema denticola (T. denticola), Fusobacterium nucleatum (F. nucleatum), Prevotella intermedia (P. intermedia), Eubacterium nodatum (E. nodatum), Eikenella corrodens (E. corrodens), Capnocytophaga spp. (Capnocytophaga spp.), Candida albicans (C. albicans), Prevotella nigrescens (P. nigrescens), Parvimonas micra (P. micra), Campylobacter rectus (C. rectus), MMP with gingival expression (gMMP)

## Data Availability

Declared none.
